# Guillain-Barré syndrome in ulcerative colitis and SARS-CoV-2 infection: a case report and literature review

**DOI:** 10.2144/fsoa-2023-0125

**Published:** 2024-05-20

**Authors:** Shema Ayadi, Rabeb Hammouga, Mohamed Slim Majoul, Hela Jamoussi, Yosra Zaimi, Asma Mensi, Mohamed Fredj, Leila Mouelhi

**Affiliations:** 1 Gastroenterology Department, Charles Nicolle University Hospital, Tunis, Tunisia; 2 Neurology Department, Charles Nicolle University Hospital, Tunis, Tunisia; 3 Faculty of Medicine of Tunis, University of Tunis El Manar, Tunis, Tunisia

**Keywords:** COVID-19, Guillain Barré syndrome, inflammatory bowel diseases, SARS-CoV-2, ulcerative colitis, venous thrombosis

## Abstract

**Aim:** Guillain-Barré syndrome (GBS) occurrence is rare during inflammatory bowel disease (IBD) and SARS-CoV-2 infection. Its association with thrombotic vascular events, which are common during these two entities, is extremely rare. **Case report:** We report an exceptional association of GBS and cerebral venous thrombosis in a 28-year-old woman with active ulcerative colitis and no previous history of SARS-CoV-2 vaccination. Mildly symptomatic SARS-CoV-2 infection was diagnosed during etiological investigations of cerebral venous thrombosis. GBS symptoms began 10 days later with clinical and electrical abnormalities consistent with axonal GBS. Other GBS causes were excluded. Favorable outcomes were noted after intravenous immunoglobulin perfusion with full recovery 12 months later. **Conclusion:** Greater attention should be focused on IBD patients with SARS-CoV-2 infection regardless of its severity.

## Plain language summary

Guillain Barré Syndrome (GBS) is a rare disorder in which the immune system attacks one's own nerves. This is responsible for progressive muscle weakness and in severe cases paralysis until death.

The association of vascular issues with GBS is rare, occurring in specific situations such as inflammatory bowel diseases or SARS-CoV-2 infections. Herein, we report an exceptional association of cerebral thrombosis and GBS, in a young female patient with ulcerative colitis and a mildly symptomatic SARS-CoV-2 infection, that was responsible for a gait disorder. Significant improvement was noted following immunoglobulin infusion and physical rehabilitation, with full recovery 12 months after treatment. Greater attention should be directed toward patients with SARS-CoV-2 infection regardless of its severity.

## Tweetable abstract

Rare but concerning: young ulcerative colitis patient developed Guillain Barré Syndrome & venous thrombosis post mild SARS-CoV-2 infection. Even benign COVID-19 cases can be deadly in some. #COVID19 #HealthComplications #IBD #LifeThreatening

## Abstract (plain language summary)

Association of Guillain-Barré syndrome (GBS) and cerebral veinous thrombosis is rare in inflammatory bowel disease. It may be triggered up by conditions like SARS-CoV-2 infection.

GBS is less frequent during ulcerative colitis than in Crohn's disease.

Axonal form of is the most frequent type of GBS in ulcerative colitis patients.

Intravenous immunoglobulins is the most common prescribed treatment of GBS with generally favorable outcomes.

The occurrence of Guillain Barré syndrome (GBS) is rare during inflammatory bowel disease and SARS-CoV-2 infection. Its association with vascular events commonly observed during these two entities, is extremely rare [1,2].

We report an exceptional association of GBS with cerebral venous thrombosis in a young female patient with active ulcerative colitis. She was treated by corticosteroids and azathioprine therapy, while experiencing pauci-symptomatic SARS-CoV-2 infection.

## Case report

A 28-year-old woman, with pancolitic ulcerative colitis and ongoing mesalazine therapy, experienced severe acute colitis. There was no relevant family medical history. Favorable outcomes were noted after first line therapy. The patient was discharged on oral corticosteroids (prednisolone 50 mg/day) and azathioprine 100 mg/day after 10 days of hospitalization.

Two weeks later, she consulted the emergency department for asthenia, abdominal pain, vomiting and liquid non-bloody diarrhea.

On physical examination, the patient had pale conjunctiva, but no clinical feature of dehydration or fever. Her blood pressure was 90/60 mmHg, heart rate of 104 beats/min and respiratory rate of 16 cycles/min. Rhythmic cardiac sounds were normal with no heart murmurs, and vesicular breath sounds were preserved. The abdomen was soft without palpable masses or organomegaly. Patient reported mild pain upon palpation of the left colic framework with no signs of peritoneal irritation. Bowel sounds were present and digital rectal examination showed normal results. Neurological examination was also normal.

Laboratory findings excluded possible azathioprine side effects as liver enzymes and lipase levels were within normal limits but with increased rate of serum CRP to 85 mg/l. White blood cells count, as well as serum creatinine and electrolytes were normal.

Colonoscopy showed presence of regenerating polyps and superficial ulcerations with healing features ruling out possible ulcerative colitis flare-up. Screening tests for *Clostridium difficile* and CMV infection were negative.

Abdominal pain and vomiting remained unexplained. Therefore, an abdominal CT scan and cerebral MRI were performed within 3 days from symptom's onset showing a thrombus of the inferior vena cava, a right surrenal hematoma, and bilateral cerebral venous thrombosis of transverse sinuses. Acquired and constitutional thrombophilia were excluded by exhaustive investigations.

Based on the evolving knowledge regarding the SARS-CoV-2 outbreak and its related thromboembolic events, a PCR test for SARS-CoV-2 was performed and reported positive. The patient had not received any SARS-CoV-2 vaccines and did not experience any respiratory complaints or need to oxygen therapy. The diagnosis of digestive manifestation of SARS-CoV-2 infection was made. Treatment with unfractionated heparin and rehydration was conducted for a few days with complete recovery.

Ten days later, the patient presented with progressive generalized weakness predominantly in the lower limbs gradually progressing to inability to walk and paraesthesia. Neurologic examination found weakness in muscular strength evaluated to 2/5 in the lower limbs and 4/5 in the upper limbs with tendon reflex abolition in both lower limbs. There was no cranial nerve involvement, dysautonomia features nor sensory deficit.

Spine MRI was normal. The patient underwent an electro-neuro-myogram 7 days after symptoms' onset revealing reduced amplitudes of action potentials especially in lower limbs and prolonged latencies of F waves without conductions blocks (Table 1). Cerebrospinal fluid (CSF) analysis was performed within 18 days from symptoms' onset. It showed a clear fluid with 1 WBC/mm^3^. CSF glucose level was 4.98 mmol/l and CSF protein level was 0.33 g/l.

**Table 1.** Values of nerve conduction.

Nerve	Sites	Side	Latency (ms)	Amplitude (mV)	Conduction velocity (m/s)	F waves (ms)
Motor						
Median	Elbow–wrist/APB	Right	3.2 (N<3.5)	4.2 (N>5)	46 (N>45)	25.3 (N<35)
		Left	3 (N<3.5)	4.6 (N>5)	48 (N>45)	26.6 (N<35)
Ulnar	Elbow–wrist/ADM	Right	3.5 (N<3.5)	4.3 (N>5)	47.5 (N>45)	28.7 (N<35)
		Left	3.2 (N<3.5)	4.2 (N>5)	46 (N>45)	29.1 (N<35)
Tibial	Knee–ankle/AH	Right	5.9 (N<5)	1.5 (N>2)	48 (N>45)	62.2 (N<55)
		Left	6.3 (N<5)	1.8 (N>2)	45 (N>45)	63 (N<55)
Peroneol	Fib head ankle/EDB	Right	5.5 (N<5)	0.8 (N>2)	42 (N>45)	68.2 (N<55)
		Left	5 (N<5)	1 (N>2)	43 (N>45)	67 (N<55)
Sensory						
Median	Wrist/3rd finger	Right	3.7 (N<3.5)	4.5 (N<5)	42 (N>45)	–
		Left	3.8 (N<3.5)	5.8 (N<5)	45 (N>45)	–
Ulnar	Wrist/5th finger	Both	Not found	Not found	–	–
Superficial peroneal	Leg/ankle	Both	Not found	Not found	–	–
Sural	Calf/ankle	Both	Not found	Not found	–	–

APB: Abductor pollicis brevis; ADM: Abductor digitiminimi; AH: Abductor hallucis; EDB: Extensor digitorum brevis; Fib head: Fibular head; N: Normal values.

Overall, clinical presentation and electrical abnormalities were consistent with axonal form of Guillain-Barré syndrome. Etiologic investigations for GBS were performed excluding viral inflammations, vitamin B12 or B1 deficiency and systemic diseases.

Intravenous immunoglobulin perfusion was prescribed for 5 days at the dose of 0.4 g/kg/day. Improvement was obtained with progressive recovery assessed by muscular testing (3/5 in lower limbs and 5/5 in upper limbs). The patient was discharged with oral corticosteroids, azathioprine, rivaroxaban and physical rehabilitation. Full functional recovery was obtained within 12 months later.

## Discussion

In this report, we present a unique case by several aspects. First, it illustrated GBS as a manifestation of SARS-CoV-2 infection in peripheral nervous system. Second, it reported a double neurologic involvement with concomitant GBS and cerebral thrombosis. Third, it presented GBS as a potential extraintestinal manifestation of ulcerative colitis.

While comparing our case to the literature, it's essential to address the practical implications for medical decision making. In our discussion, we will examine potential treatment strategies.

Additionally, we will examine how our case can guide future patient management.

Neurologic symptoms during SARS-CoV-2 infection are common, reported up to 84% of hospitalized patients [1]. The spectrum of these neurologic symptoms is wide and includes GBS as a rare manifestation. In fact, estimated GBS prevalence was about 15 cases per 100,000 SARS-CoV-2 infections and 147 reported cases to the best of our knowledge [2,3].

It's worth noting that the association of GBS and cerebral thrombosis during SARS-CoV-2 infection is exceptionally rare, with only one prior publication reporting such a case [4]. GBS occurred predominately in male patients (65.3%) [3] with a mean age of 52 years and in severely affected patients by SARS-CoV-2 infection requiring intensive care unit admission (44.9%) or mechanical ventilation (38%) [2]. However, our case stands out by an earlier age and by the fact that SARS-CoV-2 infection was relatively minor with no respiratory and general symptoms. In this case, young age is likely attributed to the association with ulcerative colitis.

The physiopathological mechanisms of GBS occurrence during SARS-CoV-2 infection are not fully understood and direct and indirect mechanisms have been proposed. Direct mechanism includes the neuroinvasive capacity of SARS-CoV-2 by ACE2 receptor activation. In fact, the coronavirus spike (S) protein attaches to ACE2 receptor for cellular entry [5]. ACE2 receptor is otherwise, present in multiple human organs, including nervous system and skeletal muscles. Indirect mechanism is represented by the autoimmune response triggered by a CRS and mediated by the inflammatory response associated with COVID-19 [3].

Clinically, the median time for GBS symptoms onset after infection is about 2 weeks, which is concordant with our case [6]. Neurological symptoms were reported in a total of 147 cases summarized in Table 2 [3]. It's important to note that muscular weakness must not be confounded with the fatigue and asthenia which are frequent during SARS-CoV-2 infection as well as myalgia, and that clinicians should be also vigilant to minor symptoms like fecal incontinence, which can be easily misdiagnosed in case of concomitant diarrhea as in our case.

**Table 2.** Neurological findings during concomitant GBS-SARS-CoV-2 infection.

Neurologic symptoms or signs	Frequency
Abnormal plantar response	7.5%
Aphasia	12.9%
Ataxia	46.3%
Dysphagia	20.4%
Facial palsy, weakness or plegia	42.2%
Faecal incontinence	4.1%
Urinary difficulties	10.9%
Hypogeusia or ageusia	17.7%
Hyporeflexia or areflexia	84.4%
Hyposmia or anosmia	15.6%
Impaired somatic sensation	72.8%
Lumbar pain	15%
Myalgia	23.8%
Neck flexion weakness	7.5%

Adapted from Bentley SA, Ahmad S, Kobeissy FH, Toklu HZ. Concomitant Guillain–Barré syndrome and COVID-19: a meta-analysis of cases. *Medicina (Kaunas)* 58(12), 1835 (2022). Doi: 10.3390/medicina58121835 with permission from [3].

GBS showed favorable outcomes with a high survival rate of 78.9%. Intravenous immunoglobulin was the most common used therapy [3].

On the other hand, IBD extra-intestinal manifestations are frequent. Peripheral nervous system involvement seems to be more frequent in UC, with a reported incidence of 1.9% [7]. However, it seems that UC presents a lower rate of demyelinating forms as compared with CD [7]. For the best of our, only ten cases of GBS and UC association in adults have been published to date knowledge (Table 3), describing GBS in all age spectrum, without sex predilection. Occurrence during active disease was more common. The axonal form of GBS is known to be more frequent in UC while demyelinating form is more prevalent in CD patients [7].

**Table 3.** Summary of published cases of association between UC and GBS compared to our case.

	Authors	Year of publication	Gender	Age at GBS Onset	Delay between UC onset and GBS	Treatment received before GBS onset	UC activity	Clinical features of GBS	Albuminocytologic dissociation	GBS electrophysiological form	GBS treatment	Outcome	Ref.
1	Torre RG *et al.*	2010	Male	74	GBS was inaugural preceded IBD onset by 2 months	None	Active with tuberculosis	Paraparesis	Absent	Axonal	IVIG	Partial improvement	[11]
2	Zimmerman J *et al.*	1985	Female	65	18 months	Sulfasalazine and oral corticosteroids	Remission	Tetraparesis Urinary disturbances Facial diplegia Dysphagia	Present	Demyelinating	Corticosteroids	Complete recovery	[12]
3			Male	58	12 years	Sulfasalazine	Remission	Areflexia Paraparesis Limb hypoesthesia Respiratory failure	Absent	Demyelinating	Corticosteroids	Complete recovery	
4	Krystallis CS *et al.*	2009	Male	59	5 years	5 aminosalicylate	Active	Limb paresthesia	Present	Demyelinating	IVIG	Complete recovery	[13]
5	Liu Z *et al.*	2018	Male	31	3 months	Mesalazine + 5 aminosalicylate	Active	Quadriparesis	Present	Axonal	IVIG	Partial improvement	[14]
6	Saito M *et al.*	1994	Female	56	NA	5 aminosalicylate	Active with Rubella infection	Quadryparesis	NA	NA	Plasmapharesis	Complete recovery	[15]
7	Roca B *et al.*	1999	Female	69	30 years	5 aminosalicylate	Remssion Mild upper respiratory infection	Quadriparesis	Present	Demyelinating	IVIG	Complete recovery	[16]
8	Tominaga K *et al.*	2019	Male	39	GBS was inaugural and preceded IBD onset by 2 weeks	Was not yet under treatment	Active	Quadriparesis Unilateral VI and VII nerve paresis	Absent	Axonal	IVIG	Complete recovery	[17]
9	Bouchra A *et al.*	2009	Female	47	10 years	5 aminosalicylate	Remission	Quadriparesis	present	Axonal	IVIG	Partial improvement	[18]
10	Jayasundara *et al.*	2022	Male	24	2 years	None	Concomittent flare-up	Paraparesis areflexia	Present	NA	IVIG	Partial improvement	[19]
11	Our case		Female	30	4 months	Azathioprine	Active with SARS-CoV-2 infection	Quadriparesis	Absent	Axonal	IVIG	Partial improvement	

IBD: Inflammatory bowel disease; IVIG: Intravenous immunoglobulin; NA: Not available; GBS: Guillain–Barré syndrome; UC: Ulcerative colitis.

The hypothesis of a common triggering factor of GBS and IBD has been proposed. Since both diseases are considered post-infectious, the role of *Campylobacter Jejuni* (*C. Jejuni*) in their respective pathogeneses has been suggested.

On one hand, large population-based cohort studies observed increased hazard rate of IBD among patients with first-time *C. Jejuni* infection compared with population comparisons [8]. This may be related to increased translocation of intestinal microflora because of loss of intestinal epithelial barrier function [9]. On the other hand, the role of *C. Jejuni* in the pathogenesis of GBS, especially the axonal form, as in our patient's case, has been suggested since 30% axonal GBS cases were preceded by *C. Jejuni* infection. Additionally, antibodies against *C. Jejuni*‘s lipo-oligosaccharides (LOS) target also myelin and axons ganglioside molecules of peripheral nerves through molecular mimicry [10].

Furthermore, a common immunological pathway of development of GBS and IBD would involve a systemic or local increase in inflammatory cytokines accompanied by Th1/Th2 imbalance. It is thought to be an extra intestinal manifestation of IBD, which is described more frequently with concomitant active disease.

However, it's important to consider potential side effects of medications used in IBD patients to manage the disease. Some drugs like Infliximab and Adalimumab known as anti-TNF-α monoclonal antibodies, have been associated with nerve-related conditions, including acute ones like GBS and chronic immune-mediated conditions [7]. In such cases, especially when active disease is present, GBS treatment can be challenging as it remains unclear whether anti-TNF-α therapy should be continued in the absence of a formal link between anti-TNF-α therapy and GBS onset. To remember, our patient had not received any prior anti-TNF-α therapy and symptoms were reported approximatively 1 month after a severe flare-up. This should also rise vigilance among clinicians regarding the possible delay of GBS occurrence while treating IBD flare-ups and especially severe flare-ups. However, clinicians should actively look for GBS symptoms while monitoring IBD flare-ups and even to educate their patients about these neurological warning signs, which can occur even after hospital discharging. It is important to note that previously reported data has not supported the possibility of SGB induced by corticosteroids or azathioprine therapy, as in our case.

Prognosis of GBS in UC patients appears favorable with no reported fatal issue. Similarly in cases of GBS complicating SARS-CoV-2 infection, intravenous immunoglobulin was the most prescribed treatment, leading to complete recovery in 6/10 of the reported cases. Our patient was treated by intravenous immunoglobulin, initially experiencing partial recovery, with full recovery achieved 12 months later.

When compared with previously published cases, our patient exhibited several common characteristics.

However, we observed a unique presentation as the association to SARS-CoV-2 infection as potential triggering factor for GBS in patient with UC has not been reported before. This association appears to trigger also, a life-threatening complication, namely cerebral venous thrombosis.

Thus, it may be reasonable to provide closer follow-up for IBD patients who contract any SARS-CoV-2 infection. An additional vigilance toward neurological signs is highly recommended. After hospital discharge or when hospitalization is not indicated, clinicians should educate their patients about potential alarming neurological signs. Importantly, they should emphasize not confusing these signs with the common symptoms of SARS-CoV-2 infection or IBD flare-ups. Clinicians should encourage their patients to seek consultation in the presence of any doubtful sign in order to promptly detect any potential GBS.

When diagnosing GBS, it may be reasonable to temporarily discontinue certain implicated therapies, such as anti-TNF therapies, and consider their reintroduction after conducting an appropriate pharmacovigilance survey.

Finally, intravenous immunoglobulin should be promptly prescribed, as it has shown favorable outcomes in the majority of published data.

## Conclusion

In summary, we reported an exceptional case of GBS in an IBD patient undergoing immunosuppressive therapy during a pauci-symptomatic SARS-CoV-2 infection. It was exceptionally associated with a severe extra pulmonary thrombosis. Greater attention should be given to patients with SARS-CoV-2 infection regardless of its severity, particularly with concomitant comorbidities like IBD and receiving immunosuppressive therapy. Clinicians should be aware of a possible delayed GBS onset following an IBD flare-up and should actively look for minor neurological symptoms when monitoring these patients to avoid misdiagnosis.

Finally, attention should be given to exclude SARS-CoV-2 infection in any patient with idiopathic GBS during the current pandemic.
